# Immune gene variation associated with chromosome-scale differences among individual zebrafish genomes

**DOI:** 10.1038/s41598-023-34467-3

**Published:** 2023-05-13

**Authors:** Sean C. McConnell, Kyle M. Hernandez, Jorge Andrade, Jill L. O. de Jong

**Affiliations:** 1 Section of Hematology-Oncology and Stem Cell Transplant, Department of Pediatrics, The University of Chicago, Chicago, IL 60637 USA; 2 Center for Research Informatics, The University of Chicago, Chicago, IL 60637 USA; 3 Present Address: Department of Medicine, Computational Biomedicine and Biomedical Data Science, Center for Translational Data Science, The University of Chicago, Chicago, IL 60637 USA; 4 Present Address: Kite Pharma, Santa Monica, CA 90404 USA

**Keywords:** Genetics, Genome, Genomics, Haplotypes, Comparative genomics, Genomic analysis, Genetic variation, Immunology, Immunogenetics

## Abstract

Immune genes have evolved to maintain exceptional diversity, offering robust defense against pathogens. We performed genomic assembly to examine immune gene variation in zebrafish. Gene pathway analysis identified immune genes as significantly enriched among genes with evidence of positive selection. A large subset of genes was absent from analysis of coding sequences due to apparent lack of reads, prompting us to examine genes overlapping zero coverage regions (ZCRs), defined as 2 kb stretches without mapped reads. Immune genes were identified as highly enriched within ZCRs, including over 60% of major histocompatibility complex (MHC) genes and NOD-like receptor (NLR) genes, mediators of direct and indirect pathogen recognition. This variation was most highly concentrated throughout one arm of chromosome 4 carrying a large cluster of NLR genes, associated with large-scale structural variation covering more than half of the chromosome. Our genomic assemblies uncovered alternative haplotypes and distinct complements of immune genes among individual zebrafish, including the MHC Class II locus on chromosome 8 and the NLR gene cluster on chromosome 4. While previous studies have shown marked variation in NLR genes between vertebrate species, our study highlights extensive variation in NLR gene regions between individuals of the same species. Taken together, these findings provide evidence of immune gene variation on a scale previously unknown in other vertebrate species and raise questions about potential impact on immune function.

## Introduction

Immune genes are among the most polymorphic genes across plant and animal genomes. This diversity helps facilitate immune protection from rapidly changing pathogens that may unpredictably attempt to evade host response. Organisms have evolved varied complements of immune genes in order to respond effectively to these threats, harnessing a wide range of unique protein families to help ensure efficient pattern recognition by the immune system^1,2^. The sequence diversity found concentrated in immune genes is often associated with positive selection and balancing selection, as populations continue to be challenged by emerging pathogens.

Adaptive and cellular immune responses are highly variable between individuals, based on extensive polymorphism, and even over time within an individual, via mechanisms such as somatic hypermutation^3^. A large subset of genes from the adaptive immune system specific to jawed vertebrates remain clustered within the Major Histocompatibility Complex (MHC) locus. In contrast to adaptive and cellular immune responses, innate and intracellular mechanisms to identify invaders are generally found to be more highly conserved, with many innate immune responses shared across plants and vertebrates^4^. For example, NOD-like receptor (NLR) genes include intracellular pattern recognition receptors (PRRs) that are mediators of direct indirect pathogen recognition and other diverse functions^5–8^. In the zebrafish, over 300 NLR genes have been annotated and found to be concentrated throughout one arm of chromosome 4^9^, making the zebrafish enriched in NLR genes compared with other vertebrates^10,11^.

Representing a key model organism for developmental biology and human disease modeling, zebrafish rely on largely the same genetic pathways as other vertebrates, including humans. Zebrafish boast a high-quality reference genome, with orthologs identified for at least 80% of human disease-related genes^12^. However, unlike other model organisms such as inbred mice, laboratory zebrafish have generally been maintained as outbred populations, with repeated introduction of fish from wild and captive-bred populations to help maximize genetic diversity^13,14^.

Previously we described divergent haplotypes of the zebrafish core MHC locus, where paralogs from the antigen processing pathway have been maintained via balancing selection for half a billion years on alternative haplotypes^15^. These haplotypes included alternate sets of immunoproteasome subunit genes and transporter associated with antigen processing (TAP) genes, as well as Class I MHC genes for antigen presentation, which had earlier been shown to have evolved into distinct complements of genes that varied markedly between individuals^16^. Building on our previous work, the goal of this study was to examine immune gene diversity throughout the zebrafish genome.

## Results

### Extensive genetic variation found in zebrafish genomes

To examine immune gene variation in the zebrafish genome, we performed deep (50–60× coverage) whole genome sequencing for two clonal zebrafish lines, CG1 and CG2, in addition to a third partially inbred individual, AB3, all derived from the AB genetic background^17^. Approximately 11 million single nucleotide variants (SNVs), and 2 million small insertions or deletions (indels) were called per individual using GATK HaplotypeCaller (Fig. S1). These raw variants were then hard filtered (Table S1) to enrich for high confidence variants, yielding 6.3–7 million SNVs per zebrafish individual. For comparison, this is substantially more than the number of SNVs (2.4–4.2 million) found in each of three different human samples (Table S2). When adjusted for genome size, the zebrafish samples much had higher SNV density, at 4.7–5.2 SNVs per kb, compared with the SNV density of 1.0–1.7 SNVs per kb in each human sample (Table 1).

**Table 1:** Summary of variant densities per genome.

Species	Sample	Genome	SNVs/kb	InDels/kb
*D. rerio*	CG2	Homozygous diploid	4.9527	1.4151
*D. rerio*	CG1	Homozygous diploid	4.7147	1.0656
*D. rerio*	AB3	Heterozygous diploid	5.2182	1.1575
*H. sapiens*	CHM	Haploid	1.0173	0.2552
*H. sapiens*	CEU	Heterozygous diploid	1.3846	0.2991
*H. sapiens*	YRI	Heterozygous diploid	1.7184	0.3289

CHM, CEU, and YRI are samples from the 1000 Genomes Project representing: a haploid complete hydatidiform mole, CHM1; Utah Resident (CEPH) with European Ancestry, NA12878; Yoruba in Ibadan, Nigeria, 19240; respectively. CG2 and CG1 are clonal zebrafish lines, and AB3 is a partially inbred fish, all on the AB genetic background.

*SNV* single nucleotide variant, *InDel* small insertion/deletion.

**Figure 1:**
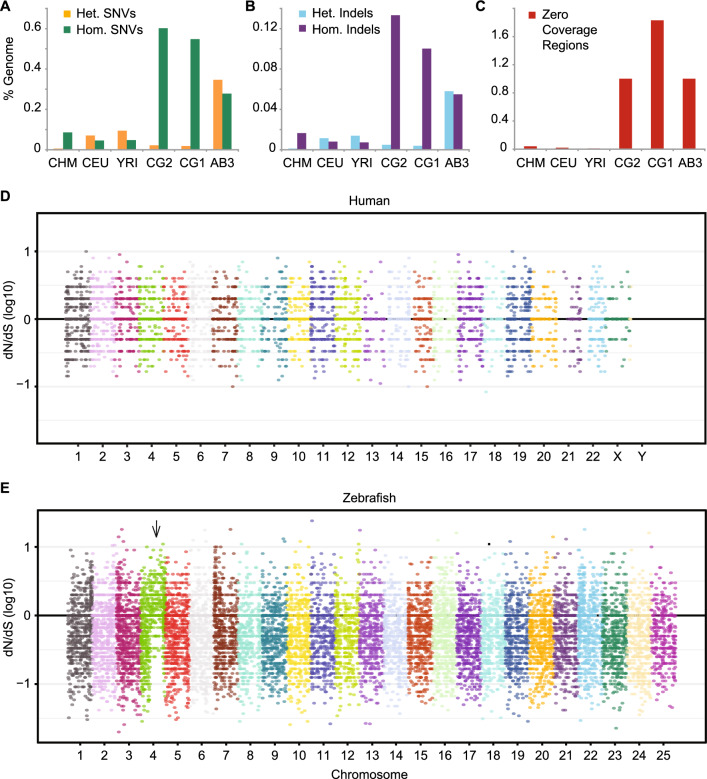
Sequence variants and evidence of positive selection. (**A**) Single nucleotide variants (SNVs) or (**B**) small insertions/deletions (Indels) were identified using GATK haplotype caller and reported as a percentage of each genome. Both heterozygous (Het.) and homozygous (Hom.) variants are shown. (**C**) Percentage of base pairs in each genome covered by Zero Coverage Regions (ZCRs), defined as no reads mapped over ≥ 2 kb intervals. Manhattan plots of the ratio of non-synonymous to synonymous mutations (dN/dS) per allele for three human (**D**) or zebrafish (**E**) individuals. Each dot represents the log10 ratio of nonsynonymous to synonymous SNVs of one gene with variants. The black horizontal line at ‘0’ indicates alleles under neutral selection, i.e. those having a dN/dS ratio of 1 (the ratio for each allele is plotted on a log10 scale). A large fraction of genes throughout the right arm of zebrafish chromosome 4 (indicated by arrow) have evidence of positive selection (dN/dS > 1). CHM, CEU, and YRI are samples from the 1000 Genomes Project representing: a haploid complete hydatidiform mole, CHM1; Utah Resident (CEPH) with European Ancestry, NA12878; Yoruba in Ibadan, Nigeria, 19240; respectively. CG2 and CG1 are clonal (homozygous diploid) zebrafish lines, and AB3 is a partially inbred fish, all on the AB genetic background.

As expected, among both SNVs (Fig. 1A) and indels (Fig. 1B), the vast majority (96–97%) of filtered variants were called as homozygous for the two clonal zebrafish lines (Table S3). Similarly, most variants were also called as homozygous (99% of SNVs and 92% of indels) for the haploid human hydatidiform mole sample CHM1, consistent with prior studies^18^. In contrast, filtered variants were more often called as heterozygous for the partially inbred AB3 zebrafish (56% of SNVs and 51% of indels), as well as for the two human samples of European and African ancestry (60–66% of SNVs and 56–68% of indels).

To examine evidence of selection pressure, we annotated filtered variants using ENSEMBL’s Variant Effect Predictor^19^ (VEP) v85. Non-synonymous (dN) and synonymous (dS) SNVs were counted per gene, across each allele among the three human and three zebrafish samples. Variants were identified in a total of 11,201 human and 19,520 zebrafish genes. Of these, 8,544 human and 18,612 zebrafish genes had synonymous variants (dS > 0), with an average of 1.4 non-synonymous and 1.6 synonymous variants per human gene, and 4.0 non-synonymous and 7.6 synonymous variants per zebrafish gene.

Evidence of positive selection was inferred for genes with a composite dN/dS ratio greater than 1 (Fig. 1D,E). 3013 zebrafish genes and 1568 human genes had evidence of positive selection. A total of 30 zebrafish genes had dN/dS ratios ≥ 10, compared with only two human genes. There is a clear trend throughout the right arm of zebrafish chromosome 4 towards increasing dN/dS (see arrow in Fig. 1E).

Similar to SNVs, relatively high numbers of small insertions and deletions (indels) were found in the zebrafish genomes. The number of indels that passed our conservative filters was 1.5–2 million per zebrafish genome, in a genome size of 1.4 Gb, for a rate of approximately 1.1–1.4 indels per kb (Fig. 1B). By comparison, the number of indels identified per human genome was 0.5–0.7 million^20^, in a genome size of approximately 3 Gb, for a rate of approximately 0.2–0.3 indels per kb (Table 1). Thus, both indels and SNVs were ~ 3 to 5 fold more abundant in the zebrafish genomes compared with the human samples analyzed.

### High density of ZCRs identified on zebrafish chromosome 4

Despite high variant density found throughout the zebrafish genome, a surprisingly large number of zebrafish genes had no variants called. Manual inspection of their sequences revealed that many of these genes lacked high quality mapped reads. This lack of aligned reads could be due to these sequences being absent from an individual zebrafish genome, or due to high divergence of these sequences relative to the reference genome.

**Figure 2:**
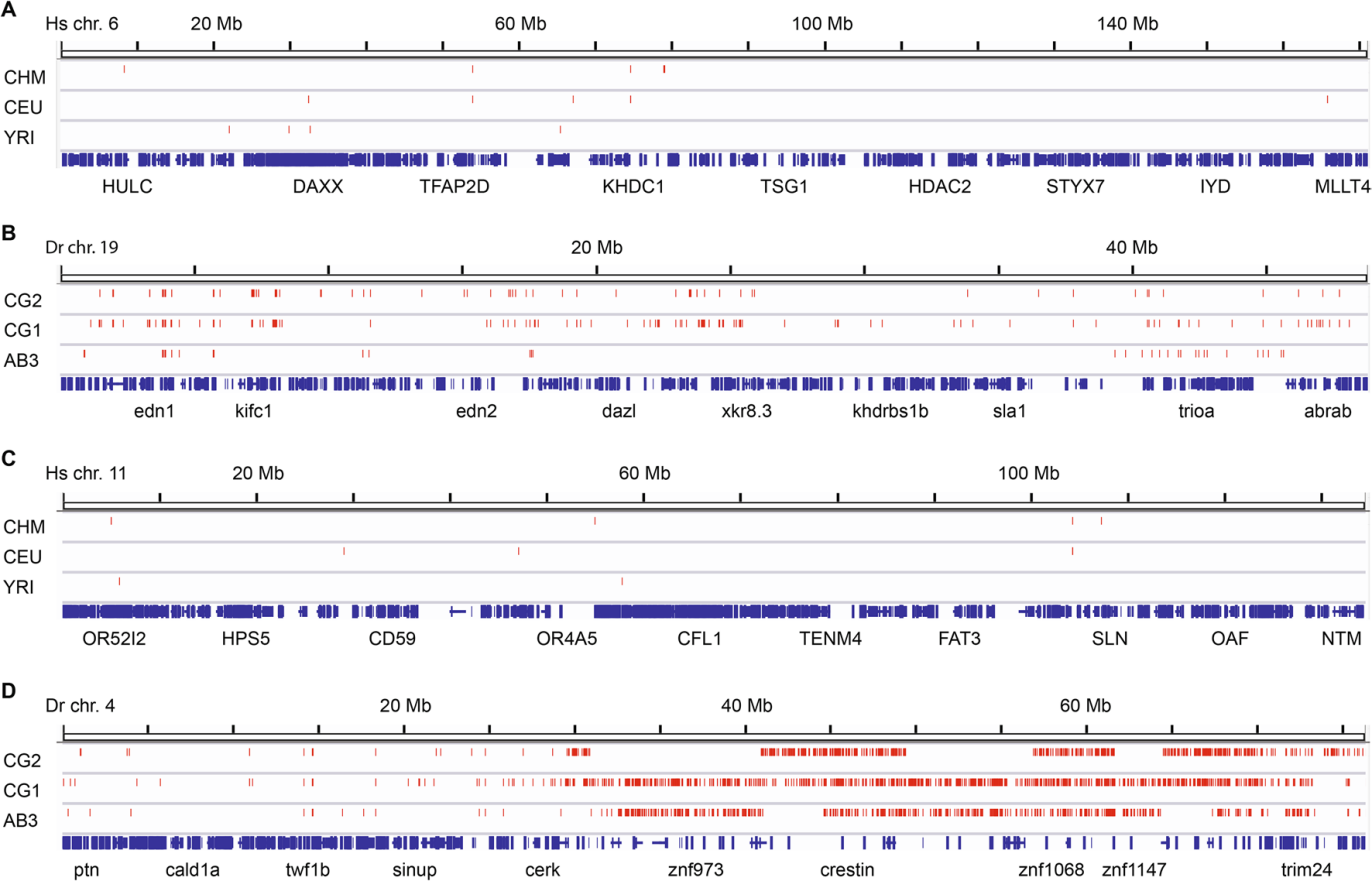
Chromosomal distribution of zero coverage regions. Comparison of (**A**) human chromosome 6 (location of the human MHC locus), (**B**) zebrafish chromosome 19 (location of the zebrafish core MHC locus), (**C**) human chromosome 11 (location of 4 out of 25 human NLR gene family members), and (**D**) zebrafish chromosome 4 (location of over 300 zebrafish NLR genes). Zero coverage regions (no mapped reads over ≥ 2 kb intervals) are displayed in red. Gene annotation is shown in blue with a small number of genes labeled. ZCRs are found more densely in zebrafish chromosomes compared with human chromosomes and a large concentration of ZCRs is distributed throughout the heterochromatic right arm of zebrafish chromosome 4 with evidence of haplotypic differences between individuals.

To help identify affected genes potentially overlooked by the variant calling pipeline, we looked for 2 kb or larger gaps without any mapped reads, representing zero coverage regions (ZCRs) previously associated with structural variation^21^. A substantially larger fraction (> 10 fold higher) of each zebrafish genome was covered in ZCRs compared with human genomes (Figs. 1C, 2). This relative prevalence of ZCRs was highlighted by unexpectedly large stretches of reference sequence with no aligned reads, offering evidence of an additional layer of variation in the zebrafish genome.

Most striking was our observation of very dense ZCRs in the late-replicating, heterochromatic arm of zebrafish chromosome 4 (Fig. 2D). This arm of zebrafish chromosome 4 has been a focus of annotation efforts that revealed a large number of immune genes, including hundreds of NLR genes^9^. Here we find that a large percentage of these NLR genes lacked mapped reads across broad expanses, in patterns that were unique to each sample, indicating divergent haplotypes between individuals. Interestingly, this arm of chromosome 4 has been identified as linked to a sex determination region that was lost during the domestication of zebrafish^22^, suggesting different selection pressure on this portion of the laboratory zebrafish genome. This region of zebrafish chromosome 4 is highly enriched for genes with evidence of positive selection (dN/dS > 1) (Fig. 1E).

### Immune genes are enriched among ZCRs and genes under positive selection

**Figure 3:**
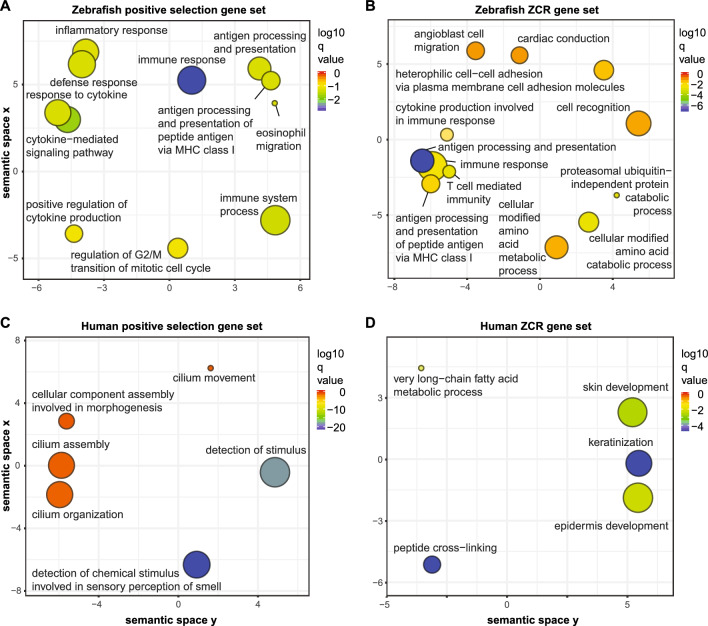
Gene annotation enrichment analysis. Genes with evidence of positive selection (dN/dS > 1) or genes with at least one exon overlapping zero coverage regions (ZCRs, without any mapped reads over ≥ 2 kb intervals) were analyzed using GO (Gene Ontology) annotation to identify genes enriched in specific biological processes. GO enrichments were summarized using REVIGO^24^. Remaining terms after adjustment for redundancy are represented as scatterplots, following semantic similarities. Bubble color indicates the log10 q-value/enrichment (see legend on right) and bubble size indicates the GO term frequency, where smaller bubbles imply more specific terms. Data are shown for zebrafish genes under positive selection (**A**), zebrafish genes overlapping ZCRs (**B**), human genes under positive selection (**C**), and human genes overlapping ZCRs (**D**). Lists of enriched pathways with genes are provided in Table S4.

To identify patterns of selection genome-wide, we performed gene pathway enrichment analysis via Gene Ontology (GO) annotation using ClusterProfiler^23^, based on combined lists of genes with evidence of positive selection (dN/dS > 1), or on combined lists of genes with coding regions overlapped by ZCRs (Table S4). These results were summarized using REVIGO^24^, which revealed that immune gene pathways including antigen processing and presentation were highly enriched among the zebrafish genes with evidence of positive selection (Fig. 3A), as well as with genes with exons overlapping ZCRs (Fig. 3B) (Table S4). In contrast, human genes associated with positive selection (Fig. 3C) or ZCRs (Fig. 3D) were significantly enriched in genes involving sensory perception, or keratinization, respectively (Table S4). Many of the human genes overlapping with ZCRs in our study have known presence/absence variation among human populations, such as *LCE3B* and *LCE3C*, which are associated with antimicrobial activity and implicated in psoriasis and wound-healing^25^. Of note, when we compared the lists of zebrafish genes overlapping ZCRs and those under positive selection (Table S4), we found only ~ 6.8% overlapped (282 shared genes), while ~ 27.7% (1153 genes) were found in the ZCR gene set only, and 65.6% (2731 genes) were only in the gene set under positive selection.

### Different patterns of ZCRs reveal unique haplotypes between individuals

**Figure 4:**
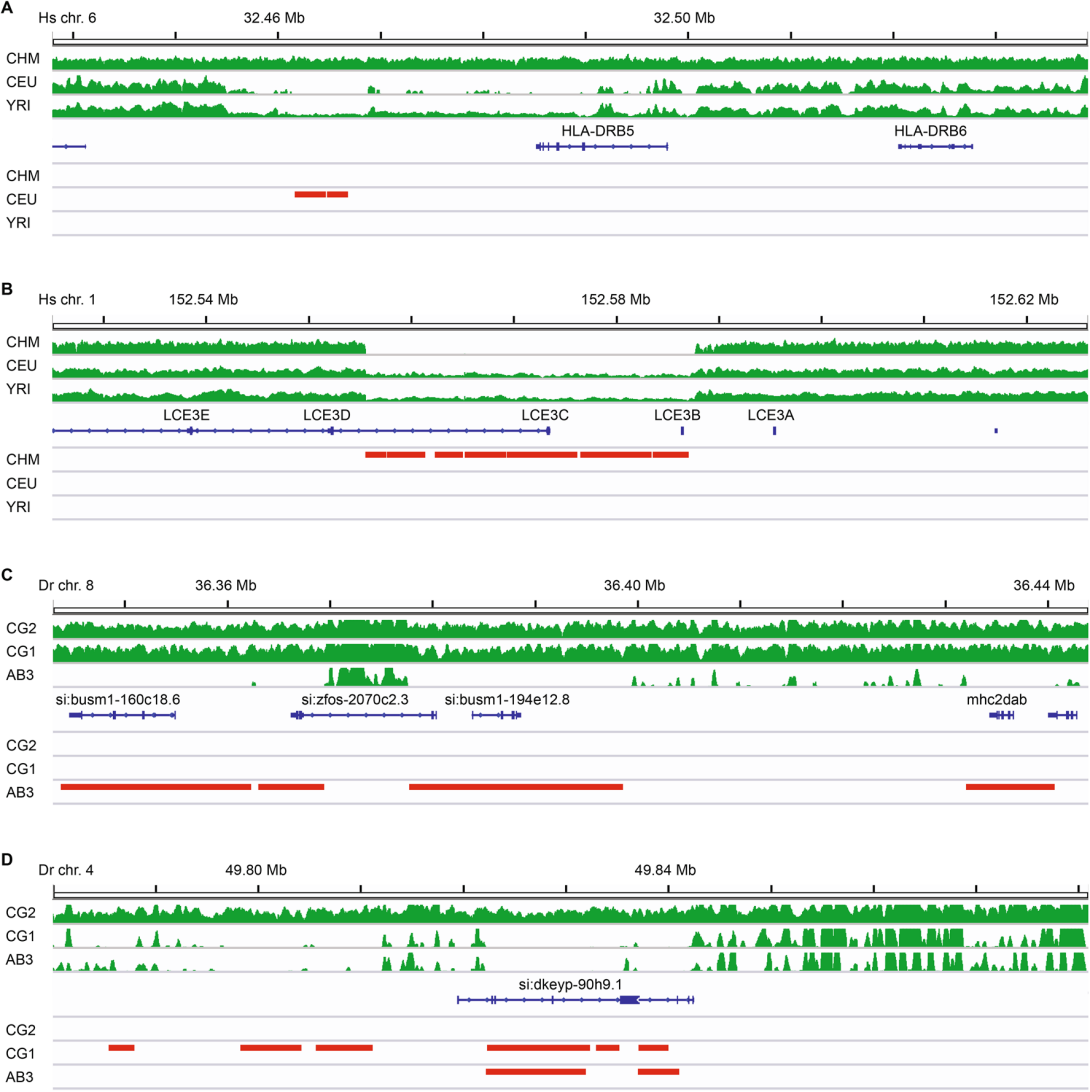
Zero coverage regions highlight unique haplotypes. Selected 100 kb region plots including (**A**) *HLA-DRB5* gene on human chromosome 6, (**B**) *LCE3C* gene on human chromosome 1, (**C**) *mhc2dab* gene on zebrafish chromosome 8, and (**D**) NLR gene (*si:dkeyp-90h9.1*) on zebrafish chromosome 4. Plots show mapped sequence read coverage across each region in green. Zero Coverage Regions (no mapped reads over ≥ 2 kb intervals) are displayed in red. Representative examples shown here were selected due to different patterns of coverage for individuals of the same species, indicating the presence of an alternative haplotype at that locus. We note similar findings indicating alternative haplotypes for additional immune gene loci throughout the zebrafish genome, including MHC Class I genes on chromosome 25 and NLR genes on chromosomes beyond chromosome 4, as highlighted in Fig. S2.

In many cases, manual inspection of zebrafish and human genes that were enriched in ZCRs revealed large, continuous regions of missing coverage, with clear boundaries. In other cases, coverage of mapped reads was more sporadic, with ZCRs unable to capture the smaller regions of missing coverage. Despite intermittent stretches of low or no coverage, exons for some human genes were narrowly missed by ZCRs, for example, MHC Class II gene *HLA-DRB5* (Fig. 4A), a gene known to have presence/absence variation. Other human genes such as *LCE3B* and *LCE3C* (Fig. 4B) overlapped ZCRs in some samples, for example, a 30 kb nearly continuous region linked to psoriasis that was identified in the CHM1 haploid genome. Our diploid human samples often had relatively low coverage over these same regions, consistent with being heterozygous for alternative haplotypes lacking reference sequence.

In contrast to human MHC genes, which appeared to lack direct overlap with ZCRs, many zebrafish MHC genes were found to overlap ZCRs, including the MHC Class II gene *mhc2dab* (Fig. 4C).

We hypothesized that ZCRs might highlight sequences that are either altogether missing, or instead highly divergent. To identify alternative haplotypes that might be associated with divergent sequences, we performed genomic assembly for the three zebrafish individuals using Discovar de novo. Analysis of these assemblies with BUSCO (Table S5) returned high percentages of the target genes for each assembly, comparable to the zebrafish reference genome, particularly for the CG1 and CG2 assemblies (85–86% complete genes). Assembly metrics (Table S6) indicated high quality assemblies including N50 values of 30–40 kb for the CG1 and CG2 assemblies and 16 kb for AB3.

Examining variation at the haplotype level, including alignment of scaffolds from our genomic assemblies, revealed that while reference or highly similar haplotypes were found across many loci, in other cases samples lacked reference haplotype sequences over large regions (Fig. S2). This was particularly evident for the zebrafish chromosome 4 region associated with NLR genes (Figs. 2D, 4D), where a highly variable patchwork of ZCRs indicated haplotypes distinct from the reference genome. Often only one of the three zebrafish samples carried reference or similar haplotypes across an NLR gene cluster.

**Figure 5:**
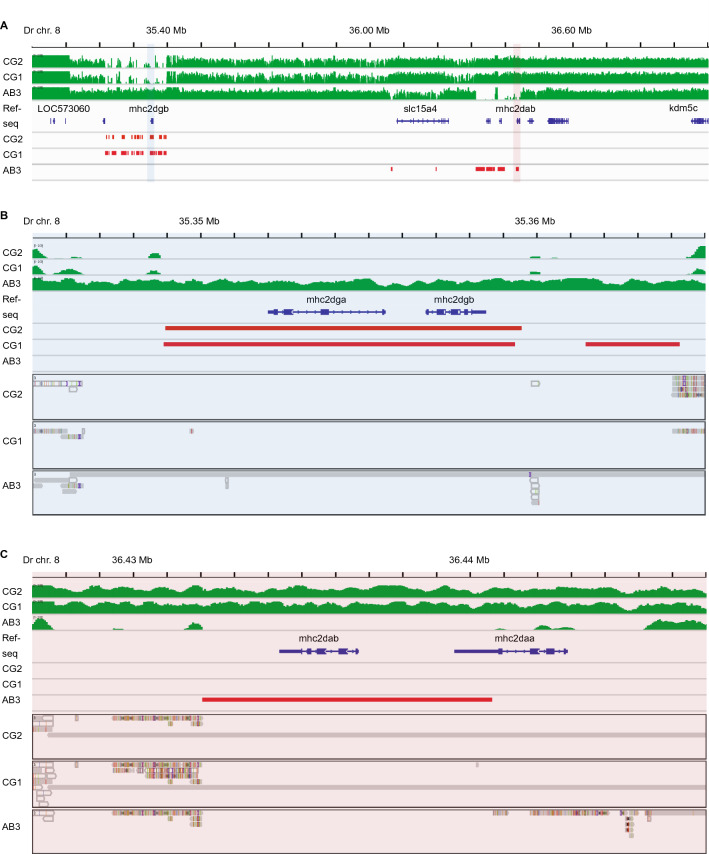
MHC Class II genes on zebrafish chromosome 8. (**A**) Read coverage across the zebrafish Class II MHC locus shows marked variability between individuals. Unlike the CG2 and CG1 fish, the AB3 zebrafish genome has a cluster of zero coverage regions (ZCRs, without any mapped reads over ≥ 2 kb intervals) in the region surrounding *mhc2dab* (highlighted in light red). In contrast, the CG2 and CG1 fish have a cluster of ZCRs in the region surrounding *mhc2dgb* (highlighted in light blue). (**B**) A detailed view of the region highlighted above in light blue (**A**) showing ZCRs overlapping the neighboring *mhc2dgb* and *mhc2dga* genes. (**C**) A detailed view of the region highlighted above in light red (**A**) showing ZCRs overlapping the neighboring *mhc2dab* and *mhc2daa* genes. Read coverage is depicted in green, ZCRs are in red, and scaffolds from Discovar assemblies that align to the reference genome are grey.

Our finding of a ZCR overlapping *mhc2dab* was somewhat unexpected, given that this gene has been considered the lone classical MHC Class II beta gene in zebrafish, and therefore might be presumed to be relatively conserved. However, we noticed a pattern in coverage throughout the larger MHC Class II locus where reads were missing over a large segment (~ 100 kb including *mhc2dab* around 36.4 Mb) for the AB3 fish. Similarly, reads were present only in the AB3 fish for an even larger region (~ 200 kb including *mhc2dgb* around 35.3 Mb) that were missing in the other fish (Fig. 5A).

BLAST searches of the CG1 and CG2 genomic assemblies returned scaffolds nearly identical to the reference sequence on chromosome 8 for *mhc2dab* (Table S7). On the other hand, for AB3 the closest scaffold match contained *mhc2dgb*, a gene with high amino acid identity to *mhc2dab* (81%), and high expression levels (Table S8). Similarly, *mhc2dga* from AB3 had high amino acid identity to *mhc2daa* (64%), and high expression, consistent with an MHC Class II classical gene signature.

Thus, only the AB3 fish had coverage data and genomic scaffolds consistent with reference sequence that encompassed *mhc2dga* and *mhc2dgb* (Fig. 5B). On the other hand, only the CG1 and CG2 fish had coverage data and genomic scaffolds including *mhc2dab* and *mhc2daa* (Fig. 5C). This pattern is reminiscent of our earlier observation of alternative haplotypes for the MHC Class I locus^15^, providing evidence that two alternative MHC Class II haplotypes may be included within the zebrafish reference genome, assembled in tandem as a composite haplotype. The findings reported in Figs. 4, 5 and S2 are representative examples and not isolated findings. We have noted similar results of ZCRs associated with other immune gene loci, including a substantial portion of the remaining known MHC and NLR gene loci throughout the zebrafish genome on chromosomes 1, 3, 8, 13, 19, and 22 (data not shown).

### Presence/absence variation affects most zebrafish MHC and NLR genes

**Figure 6:**
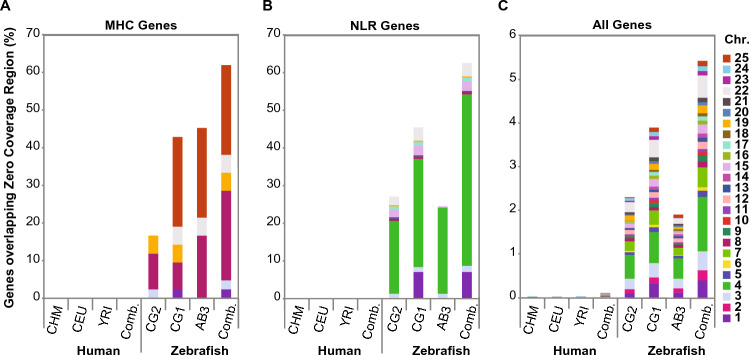
MHC and NLR genes associated with zero coverage regions (ZCRs). The percentage of (**A**) major histocompatibility complex (MHC) genes, (**B**) NOD-like receptor (NLR) genes, or (**C**) all genes in each of three human or zebrafish genomes, with at least one exon overlapping ZCRs. ‘Comb.’ refers to the combined list of genes from all three individuals that overlaps with ZCRs. CHM, CEU, and YRI are human samples from the 1000 Genomes Project representing: a haploid complete hydatidiform mole, CHM1; Utah Resident (CEPH) with European Ancestry, NA12878; Yoruba in Ibadan, Nigeria, 19240; respectively. CG2 and CG1 are clonal zebrafish lines, and AB3 is a partially inbred fish, all on the AB genetic background (the gene lists used for (**A,B**) are found in Table S9. Total number of zebrafish MHC genes = 42; total number of human MHC genes = 37. Total number of zebrafish NLR genes = 368^9^. The data used to make the bar graphs are found in Table S11).

Because pathway analysis implicated zebrafish immune genes as highly enriched among genes with evidence of positive selection and genes associated with ZCRs, we elected to examine association with MHC and NLR genes more comprehensively. We used custom gene lists (Table S9) because these genes often lacked RefSeq annotation. Strikingly, 62% of the MHC gene set (Fig. 6A) and 63% of the NLR gene set (Fig. 6B) were associated with ZCRs in at least one of the three zebrafish samples, compared with 0% for MHC and NLR genes in humans. When taking all genes into consideration, 5% (n = 1461) of zebrafish genes had exons overlapping with ZCRs (Fig. 6C, Tables S10 and S11), while less than 0.2% (n = 36) of all human genes overlapped ZCRs in at least one sample. This high level of presence/absence variation in zebrafish individuals is expected to disproportionately affect immune function given the large number of immune genes involved.

## Discussion

Zebrafish genomes carry much higher levels of variation than human genomes^13,26–28^. This was evident even when comparing homozygous diploid (effectively double haploid) clonal fish genomes to human genomes carrying relatively high levels of variation, for example the genome of an individual of African ancestry. However, our analysis likely still represents an underestimate of variation due to challenges in characterizing divergent zebrafish gene loci, including under-sampling, our focus on homozygous diploid individuals, and inherent limitations of reference genomes.

Due to challenges associated with using CNV-calling pipelines for zebrafish sequencing data, we chose ZCRs (to highlight regions with no mapped reads) to identify putative homozygous deletional CNVs. Our findings suggest that while ZCRs were highly specific to identify larger regions with missing coverage, they were not sensitive to detect all regional polymorphism in genes. This was particularly evident for apparent heterozygous deletions or when read coverage was more highly variable, due in part to mapping of closely related gene sequences.

ZCRs are thus overall likely to underestimate the degree of structural variation throughout these genomes. This includes alternative haplotypes which will likely require long-read sequencing or similar approaches to fully resolve^29^. However, we note that even after solving the challenges of ensuring that these repetitive stretches of sequence are well-resolved^30,31^, annotated^32^, and characterized^33,34^, a single reference genome sequence may fundamentally be incapable of capturing the diversity of these alternative haplotypes.

This may be particularly relevant for immune genes, as our gene pathway analysis showed that much of the zebrafish variation was concentrated in genes associated with immune function. MHC genes, arguably the most polymorphic genes in humans, also exhibited variability in zebrafish genomes that far exceeded MHC variation found in humans (Fig. 6A). Even nonclassical MHC genes, which are largely monomorphic in humans, were also found to be highly polymorphic in zebrafish, consistent with more widespread differences in immune genes between individuals. Such extensive variation in zebrafish immune genes suggests the possibility of a different mechanism for generating variation in zebrafish genomes compared to humans, a question that is worthy of further study.

On chromosome 4, which is highly enriched for NLR genes^9^, we found that roughly half of the chromosome appeared largely different from one individual fish to the next. The magnitude of this variation was particularly striking for the CG1 fish, where reads across nearly half of chromosome 4 failed to align to the reference genome. Yet, most of these segments (e.g., ~ 1 Mb blocks) still had high sequence similarity to the reference genome in one or more of the other fish, indicating that our alignment approach worked well for the sequences present in these other samples. Poorly mapped or missing reads, as outlined by ZCRs, were concentrated throughout these large segments of chromosome 4 that varied markedly between individuals. Our genomic assemblies for the individual zebrafish provide additional evidence that in many cases these poorly mapped reads were due to markedly divergent sequences (or alternatively, presence/absence variation) and not due to low quality sequence data.

Assemblies for clonal fish lines CG1 and CG2 should aid experiments designed for animals that are nearly genetically identical, with some expected genetic drift, analogous to experiments with inbred mouse strains^35^. On the other hand, individuals from traditional outbred zebrafish lines should be expected to be separated by more significant genetic differences, particularly at immune loci. This variation is likely to complicate some experiments in outbred zebrafish including measurements of immune response.

Despite annotation efforts to define the scope of the NLR genes in the reference genome^9^, further work is needed to uncover additional genes in alternative haplotypes that we identified, each spanning up to 20 Mb. These genes were underreported in our assessment of positive selection, due to annotation being incomplete. Analysis of the genes in these ZCR regions using RNA-Seq data across different tissues would clarify expression patterns, provide insight into their function, and further improve annotation to include genes that may not be present in current reference genome sequences. Some zebrafish NLR genes have already been implicated in immune response^36–39^.

The functional implications of such expanded and diverse repertoires of NLR genes, along with any consequences for evolution of the host genome, remain interesting topics for further study. Species can gain distinctive collections of immune genes, which allow them to respond to the evolving threats of pathogens. Some immune genes have been found to segregate within only certain individuals within a species^40,41^, including in zebrafish^16^. While previous studies of vertebrate NLR genes have focused on differences between species^10,11,42–45^, here we find that the proliferation of zebrafish NLR genes appears highly variable between individuals.

NLR genes have undergone multiple, independent expansions throughout jawed vertebrate evolution, which is thought to be tied to the immunological function of NLR genes^44,46^. Intriguingly, plant NLRs also maintain strain-specific complements of NLR genes, which are known to help mediate strain-specific pathogen resistance^5,6^. While the functional roles of these highly variable zebrafish NLR gene sets remain unclear, they may also be anticipated to help mediate strain-specific pathogen resistance.

## Conclusions

Genomic variation including SNVs, indels and ZCRs, was more abundant in zebrafish genomes compared with human genomes. Immune genes were enriched among genes overlapping ZCRs and genes under positive selection in zebrafish. Highly divergent haplotypes were identified at immune gene loci, including the MHC Class II locus, and most notably throughout one arm of chromosome 4 associated with NLR genes. To our knowledge this scale of immune gene diversity between individuals of the same species, where hundreds of genes may vary markedly between individuals including across half of a vertebrate chromosome, has not previously been described in vertebrates. In addition to their potential impact on immune function, these divergent loci also offer a unique opportunity to study mechanisms driving large-scale genome variation and evolution.

## Methods

### Zebrafish

The golden-derived clonal lines, CG1^47^, and CG2^48^, were each generated through two rounds of parthenogenesis and generously provided by Dr. Sergei Revskoy. The AB3 individual zebrafish from the AB zebrafish strain was also kindly provided by Dr. Sergei Revskoy. One individual male animal of each strain was selected at random from a tank of healthy adults approximately one year of age. Zebrafish husbandry, care and all experiments were performed in accordance with the Guide for the Care and Use of Laboratory Animals^49^ and as approved by the University of Chicago Institutional Animal Care and Use Committee. All methods are reported in accordance with ARRIVE guidelines.

### Genomic sequencing

To isolate genomic DNA, each individual adult zebrafish was euthanized and placed in proteinase K digestion buffer overnight, followed by phenol chloroform extraction and ethanol precipitation, using previously described methods^50^. Prior to genomic sequencing, carryover organics were removed from the genomic DNA using the DNeasy Blood & Tissue kit (Qiagen) according to the manufacturer’s instructions. We used a single-library per sample approach for high-throughput sequencing. Briefly, Illumina TruSeq DNA PCR-free libraries were constructed from genomic DNA isolated from each individual zebrafish. To facilitate Discovar de novo assemblies, the libraries were individually sequenced in single lanes on a HiSeq2500 instrument (Rapid run mode), using paired-end 2 × 250 bp reads, providing approximately 50–60 × coverage.

### Read alignment

Zebrafish raw reads were aligned to the GRCz10 assembly (Illumina iGenomes: https://support.illumina.com/sequencing/sequencing_software/igenome.html) using BWA aln v0.7.12^51^ with aln parameters: *-q 5 –l 32 –k 2 –o 1*; sample parameters: *-a 1350* and formatted using sambamba v0.5.9^52^.

### SNV/indel detection

D. rerio alignments were filtered to remove unaligned reads and alignments with low mapping quality (MAPQ > 10) using sambamba. Filtered alignments were base quality recalibrated using GATK v3.6.0^53^. Filtered and quality recalibrated alignments were used to detect genotypes using the GATK HaplotypeCaller and GenotypeGVCFs tools. To call genotypes, haplotypes were first detected in each sample separately then joint-genotyping was performed across all three samples using the GATK HaplotypeCaller/GenotypeGVCFs. Raw genotypes were hard filtered to remove low quality calls and potential artifacts using GATK’s SelectVariants and VariantFiltration (Table S1). Basic variant metrics were extracted using RTG Tools v3.7.1^54,55^ and custom scripts. Filtered variants were annotated using the ENSEMBL’s VariantEffectPredictor (VEP) v85^19^ with RefSeq cache version 85.

### dN/dS analysis

VEP annotations were processed to select the mutational impact on the canonical transcript for each alternate allele. Synonymous and non-synonymous effects were then counted for each gene based on the canonical transcript and imported into R. The ratio of non-synonymous to synonymous counts (dN/dS) for each gene was estimated. Genes with dN/dS > 1.0 were used for an enrichment analyses with the clusterProfiler v3.2.15^23^ and DOSE v3.0.10^56^ R BioconductoR packages.

### Human sample coverage and variant data

Publicly available human genomic sequencing files were obtained from the 1000 Genomes Project^20^, including Utah Resident (CEPH) with European Ancestry, NA12878; Yoruba in Ibadan, Nigeria, 19240; and a haploid complete hydatidiform mole, CHM1. All samples in the 1000 Genomes Project were obtained following the ethical guidelines of the Ethical Legal and Social Implications (ELSI) Group and informed consent was obtained from all participants. The use of these de-identified samples was exempt from oversight by the University of Chicago Institutional Review Board.

These human alignment files are publicly available and were downloaded from the 1000 Genomes FTP site:

/1000genomes/ftp/phase3/data/NA12878/high_coverage_alignment/NA12878.mapped.ILLUMINA.bwa.CEU.high_coverage_pcr_free.20130906.bam.

/1000genomes/ftp/phase3/data/NA19240/high_coverage_alignment/NA19240.mapped.ILLUMINA.bwa.YRI.high_coverage_pcr_free.20130924.bam.

/vol1/ftp/technical/working/20150612_chm1_data/alignment/150140.mapped.ILLUMINA.bwa.CHM1.20131218.bam.

The human VCF files are also publicly available and were downloaded from: ftp://ftp-trace.ncbi.nih.gov/1000genomes/ftp/technical/working/20140625_high_coverage_trios_broad/; ftp://hengli-data:lh3data@ftp.broadinstitute.org/hapdip/vcf-flt/CHM1.mem.hc-3.3.flt.vcf.gz.

### Zero coverage region (ZCR) analysis

Unfiltered BAM files were converted to 1x-coverage bigWig files using deeptools v2.4.3^57^. Gap regions were extracted from the UCSC table browser and removed from the bigWig files using bwtool v1.0-gamma^58^. Regions from gap-removed bigWig files with 0 coverage were extracted and converted to BED files using bwtool and those ≥ 2 kb in length were extracted for downstream analysis. The selected regions were intersected with GTF files and the genes with at least one exon overlapping were extracted using the pybedtools v0.7.9 python package^59,60^ and custom scripts. Genes with overlapping ZCRs were then used for enrichment analyses in a similar manner as the dN/dS analysis.

### Genomic assemblies generated using Discovar de novo

Raw reads were converted to the unmapped bam format using Picard tools (2.2.1; http://broadinstitute.github.io/picard/). Discovar de novo^61^ was used to generate genomic assemblies with default settings (build 52488; https://www.broadinstitute.org/software/discovar/blog/). While the Discovar de novo assemblies were each generated independently of the reference genome, the GRCz10 zebrafish assembly (version 140) was subsequently referenced for the purposes of scaffold mapping.

### BUSCO assembly metrics

Discovar de novo assemblies were analyzed using BUSCO (Benchmarking Universal Single-Copy Orthologs) (build v1.22 depending on Augustus v 3.1, blast + 2.2.31, and hmmer3.1b2; http://busco.ezlab.org/), modified to run tblastn outside of the BUSCO script. The BUSCO approach provides quantitative assessment of genome quality by assessing genome completeness, based on an evolutionarily conserved list of 3023 vertebrate single-copy orthologs^62^. Because we found that the tBLASTn results were sometimes incomplete using the implementation provided by the BUSCO genome assemblies, we instead performed our own tBLASTn searches on our genome assemblies using a separate installation. Complete tBLASTn results for each of our genome assemblies were then returned to BUSCO for gene prediction and assessment of completeness. We also included the GRCz10 reference genome in this modified BUSCO pipeline for comparison.

## Supplementary Information

Supplementary Information.

Supplementary Table S4.

Supplementary Table S11.

## Data Availability

The datasets generated and/or analyzed during the current study are included in this published article (and its supplementary information files) or are available from the following repositories. Genomic assembly data generated in this study have been submitted to the NCBI BioProject database (https://www.ncbi.nlm.nih.gov/bioproject/) under accession numbers PRJNA292113, LKPD02000000 (CG2); PRJNA454110, JALCZS000000000 (CG1); and PRJNA454111, JALCZT000000000 (AB3). Raw sequence data have been deposited in the NCBI short read archives (SRA) with accession numbers SRR7080552, SRR7081528, and SRR7081557. Supplemental data files, including bigWig and BED files have been published in the CyVerse Data Commons under https://de.cyverse.org/data/ds/iplant/home/shared/commons_repo/curated/McConnell_ZeroCoverageRegions_2022.
