# Supplementary material for: Immune gene variation associated with chromosome-scale differences among individual zebrafish genomes
Source: Sci Rep. 2023 May 13;13:7777. doi: 10.1038/s41598-023-34467-3 (PMC10183018; doi:10.1038/s41598-023-34467-3)
Supplement: Supplementary file 1 — Supplementary Information. [file 41598_2023_34467_MOESM1_ESM.pdf]

Supplemental file for:

Immune gene variation associated with chromosome-scale differences among individual zebrafish genomes

Sean C. McConnell, Kyle M. Hernandez, Jorge Andrade, and Jill L.O. de Jong

Table of Contents for Supplementary Materials

Page

1 Figure S1. Flowchart of steps from raw sequencing data to variant detection and annotation.

2 Figure S2. Examples of genes with zero coverage regions (ZCRs) demonstrating distinct haplotypes

3 Table S1. List of filters applied to zebrafish raw variants to enrich for higher confidence variants, applied at the locus level.

4 Table S2. Number of known (Ensembl dbSNP 142), novel, and combined (all) variants remaining per genome, after filtering for higher confidence variants.

5 Table S3. Ratios of heterozygous (Het.) to homozygous (Hom.) variants for known (Ensembl dbSNP 142), novel, and combined (all) variants remaining per genome, after filtering for higher confidence variants.

6 Table S5. BUSCO analysis of genomic assemblies.

7 Table S6. Overview of assembly metrics.

8 Table S7. Genomic scaffolds associated with 521 zebrafish chromosome 8.

9 Table S8. Nomenclature and expression for zebrafish MHC class II genes.

10 Table S9. Lists of Zebrafish and Human MHC genes, and Human NLR genes.

13 Table S10. Summary of ZCR regions and number of genes found overlapping with ZCRs for the zebrafish and human genomes.

Separate Excel files

Table S4. (Table S4.xlsx) Lists of zebrafish and human genes associated with positive selection (dN/dS ratio >1), and genes found to be overlapping with zero coverage regions (ZCRs)

Table S11. (Table S11.xlsx) Summary of MHC, NLR, and all genes found overlapping with ZCRs for the zebrafish and human genomes.

Figure S1

Flowchart of steps from raw sequencing data to variant detection and annotation.

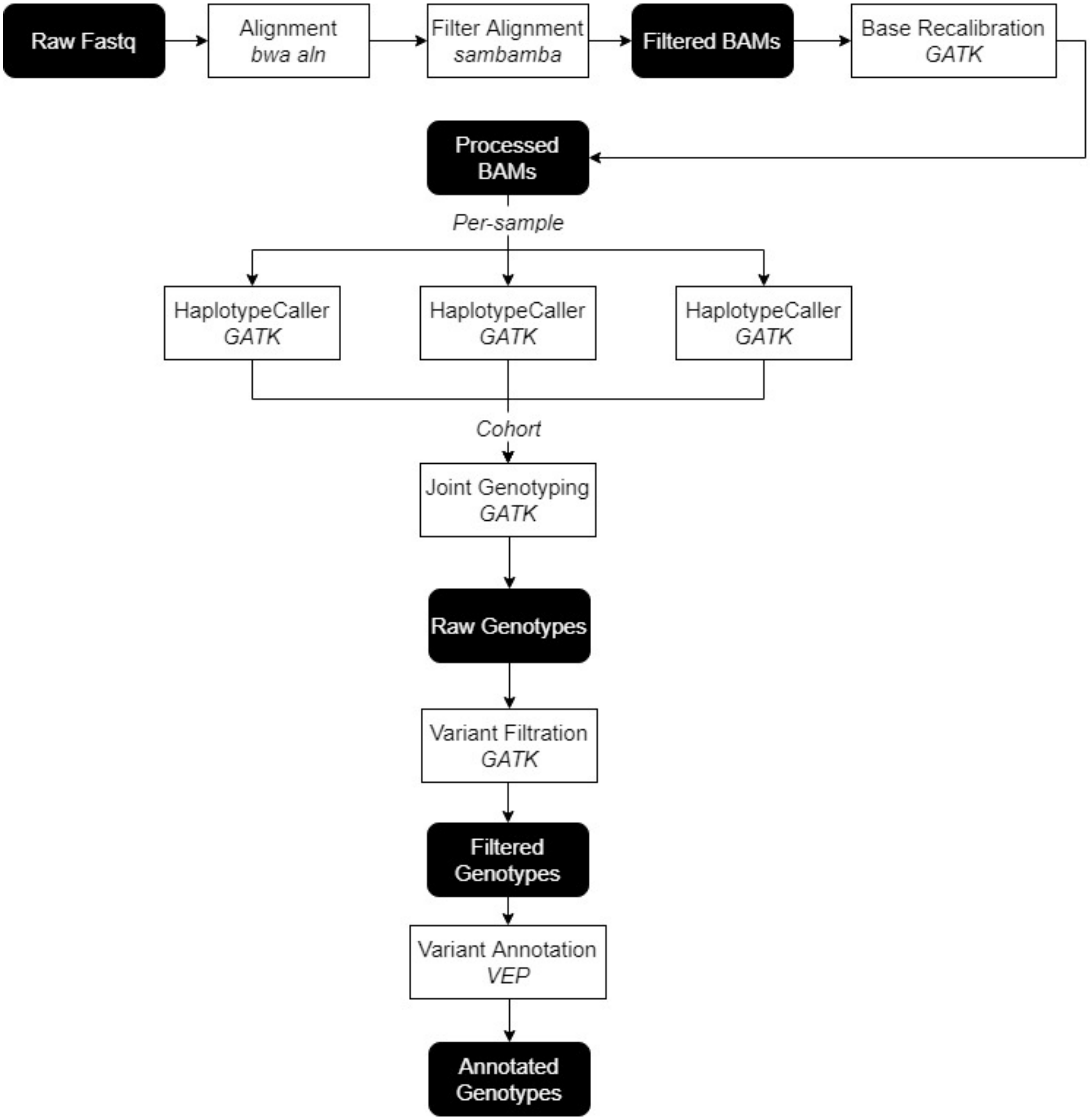

Figure S2

A. MHC Class I L lineage genes on zebrafish chromosome 25.
Unlike the CG2 fish, the CG1 and AB3 zebrafish genomes have a cluster of ZCRs (no coverage of aligned reads over 2kb) overlapping several Class I genes in a region ~150 kb surrounding mhc1lja. In contrast to the CG1 and AB3 fish, Discovar assembly scaffolds for the CG2 genome align to this region and their sequences have high percent identity to the reference genome. ZCRs were frequently found associated with MHC genes, including other MHC genes not found in this large cluster of MHC genes on chromosome 25. Read coverage is depicted in green, zero coverage regions (ZCRs) in red, NLR genes annotated by Howe et al. (2016) in purple, and scaffolds from the Discovar assemblies aligned to the reference genome in grey.

B. NLR gene cluster on zebrafish chromosome 1.
The CG1 zebrafish genome has a high density of ZCRs (no coverage of aligned reads over 2kb) in this region. In contrast to the CG1 fish, Discovar assembly scaffolds for the CG2 and AB3 genomes align to this region and their sequences have high percent identity to the reference genome. Read coverage for AB3 was lower, consistent with this fish being heterozygous for reference sequence. ZCRs were frequently found associated with NLR genes on several additional chromosomes outside of the large cluster tiled across one arm of zebrafish chromosome 4.

A

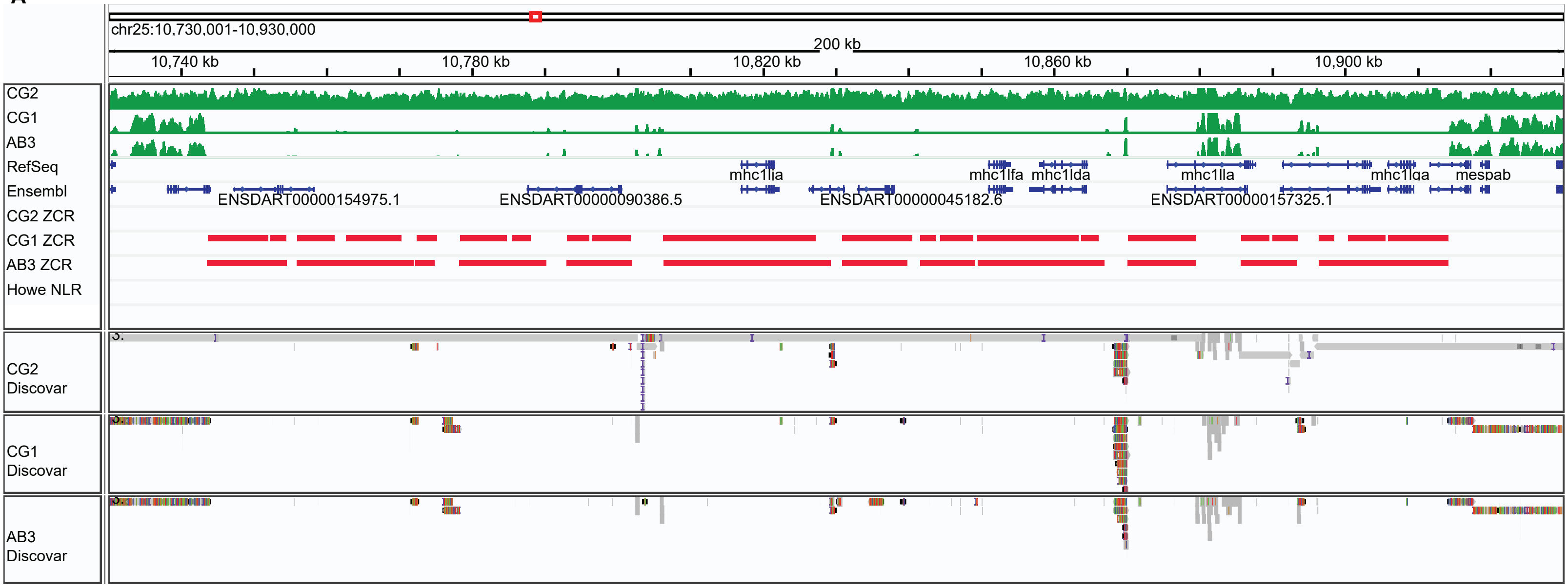

B

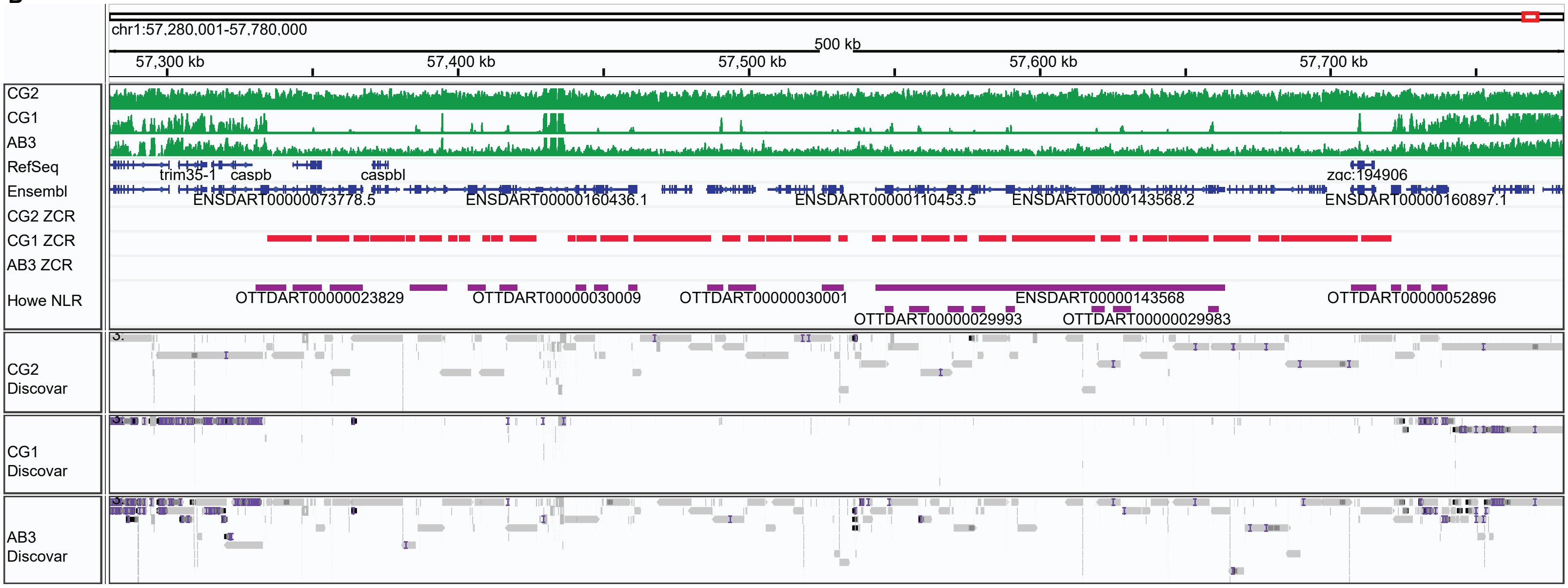

Table S1

List of filters applied to zebrafish raw variants to enrich for higher confidence variants, applied at the locus level.

Type	Filter	Description	Type
SNP	LowQD	QD < 2.0	locus
SNP	LowMQ	MQ < 40.0	locus
SNP	StrandBias	FS > 60.0	locus
SNP	HighSOR	SOR > 4.0	locus
SNP	LowMQRankSum	MQRankSum < -12.5	locus
SNP	LowReadPosRankSum	ReadPosRankSum < -8.0	locus
**Type**	**Filter**	**Description**	**Type**
INDEL	LowQD	QD < 2.0	locus
INDEL	StrandBias	FS > 200.0	locus
INDEL	HighSOR	SOR > 1.0	locus
INDEL	LowReadPosRankSum	ReadPosRankSum < -20.0	locus

Table S2

Number of known (Ensembl dbSNP 142), novel, and combined (all) variants remaining per genome, after filtering for higher confidence variants.

CHM, CEU, and YRI are samples from the 1000 genomes project. CHM is a haploid complete hydatidiform mole, CHM1. CEU is a Utah resident (CEPH) with European Ancestry (NA12878). YRI is person of the Yoruba community in Ibadan, Nigeria, (19240). CG2 and CG1 are clonal zebrafish lines, and AB3 is a partially inbred zebrafish, all on the AB genetic background.

Sample	Set	SNVs (n)	Insertions (n)	Deletions (n)	Insertion/ Deletion ratio
CG2	known	4005069	5596	303765	0.02
CG1	known	3935546	3714	265239	0.01
AB3	known	4065092	3998	265892	0.02
CG2	novel	2633746 (39.7%)	930670 (99.4%)	656462 (68.4%)	1.42
CG1	novel	2384250 (37.7%)	693058 (99.5%)	465989 (63.7%)	1.49
AB3	novel	2929636 (41.9%)	748935 (99.5%)	529171 (66.6%)	1.42
CG2	all	6638815	936266	960227	0.98
CG1	all	6319796	696772	731228	0.95
AB3	all	6994728	752933	795063	0.95
**Sample**	**Set**	**SNVs (n)**	**Insertions (n)**	**Deletion (n)**	**Insertion/ Deletion**
CHM1	known	2447054	235762	276605	0.85
CEU	known	3377708	268821	304765	0.88
YRI	known	4181956	279183	324387	0.86
CHM1	novel	47250 (1.89%)	54352 (18.7%)	58678 (17.5%)	0.93
CEU	novel	17381 (0.51%)	57333 (17.6%)	58086 (16.0%)	0.99
YRI	novel	31477 (0.75%)	59151 (17.5%)	85477 (20.9%)	0.69
CHM1	all	2494304	290114	335283	0.87
CEU	all	3395089	326154	362851	0.9
YRI	all	4213433	338334	409864	0.83

Table S3

Ratios of heterozygous (Het.) to homozygous (Hom.) variants for known (Ensembl dbSNP 142), novel, and combined (all) variants remaining per genome, after filtering for higher confidence variants.

Sample	Set	SNP Het./H0om. ratio	Insertion Het./Hom. ratio	Deletion Het./Hom. ratio
CG2	known	0.01 (42649 /3962420)	0.01 (60 /5536)	0.01 (2921 /300844)
CG1	known	0.01 (39278 /3896268)	0.02 (74 /3640)	0.01 (2867 /262372)
AB3	known	1.05 (2078827 /1986265)	0.90 (1894 /2104)	0.88 (124662 /141230)
CG2	novel	0.08 (189220 /2444526)	0.03 (30033 /900637)	0.06 (34491 /621971)
CG1	novel	0.07 (160228 /2224022)	0.04 (23733 /669325)	0.06 (25970 /440019)
AB3	novel	1.75 (1864812 /1064824)	1.04 (382405 /366530)	1.18 (286181 /242990)
CG2	all	0.04 (231869 /6406946)	0.03 (30093 /906173)	0.04 (37412 /922815)
CG1	all	0.03 (199506 /6120290)	0.04 (23807 /672965)	0.04 (28837 /702391)
AB3	all	1.29 (3943639 /3051089)	1.04 (384299 /368634)	1.07 (410843 /384220)
**Sample**	**Set**	**SNP Het./Hom. ratio**	**Insertion Het./Hom. ratio**	**Deletion Het./Hom. ratio**
CHM1	known	0.01 (19743 /2427311)	0.05 (10327 /225435)	0.05 (13640 /262965)
CEU	known	1.52 (2036311 /1341397)	1.17 (144880 /123941)	1.39 (177232 /127533)
YRI	known	1.94 (2760962 /1420994)	1.49 (166901 /112282)	1.78 (207559 /116828)
CHM1	novel	0.55 (16757 /30493)	0.13 (6227 /48125)	0.24 (11532 /47146)
CEU	novel	5.07 (14517 /2864)	2.12 (38937 /18396)	2.98 (43478 /14608)
YRI	novel	6.25 (27136 /4341)	3.81 (46854 /12297)	5.11 (71478 /13999)
CHM1	all	0.01 (36500 /2457804)	0.06 (16554 /273560)	0.08 (25172 /310111)
CEU	all	1.53 (2050828 /1344261)	1.29 (183817 /142337)	1.55 (220710 /142141)
YRI	all	1.96 (2788098 /1425335)	1.72 (213755 /124579)	2.13 (279037 /130827)

Table S5

BUSCO analysis of genomic assemblies. GRCz10 represents a chromosome-level assembly for the zebrafish reference genome. Analysis was restricted to scaffolds at least 1kb in size. To improve performance, BUSCO was customized by independent tblastn. The numbers of Complete genes (Complete single-copy or Complete duplicated; C), Fragmented genes (Frag.; F), and Missing genes (M) are shown, along with their percentages. The total number of genes analyzed was 3023.

Sample	Assembly	Complete	Complete single-copy	Complete and duplicated	Frag.	Missing	BUSCO benchmark
Tuebingen	GRCz10 (chr.)	2589	2464	125	251	183	C:85%[D:4.1%],F:8.3%, M:6.0%
CG2	SOAP (v1.0)	2594	2480	114	238	191	C:85%[D:3.7%],F:7.8%, M:6.3%
CG2	Discovar (v2.0)	2603	2485	118	239	181	C:86%[D:3.9%],F:7.9%, M:5.9%
CG1	Discovar (v2.0)	2618	2504	114	237	168	C:86%[D:3.7%],F:7.8%, M:5.5%
AB3	Discovar (v2.0)	2368	2260	108	309	346	C:78%[D:3.5%],F:10%, M:11%

Table S6

Overview of assembly metrics. GRCz10 represents a chromosome-level assembly for the zebrafish reference genome. For each assembly, the N50 was defined as the length of the shortest scaffold at 50% of total genome length. In contrast, the L50 count was defined as the smallest number of scaffolds whose length sum comprises half of the genome size. N's were not considered for the %GC calculation.

Sample	Assembly	Length (bp)	N50 (bp)	L50 (#)	%GC	Ns/100kb	Scaf. (#)
CG2	SOAP (v1.0)	1,228,709,066	34,289	9,949	36.15	5766.12	73,507
CG2	Discovar (v2.0)	1,290,936,762	40,494	8,876	36.36	120.35	83,098
CG1	Discovar (v2.0)	1,287,273,931	38,614	9,213	36.36	124.31	85,796
AB3	Discovar (v2.0)	1,498,361,605	16,522	20,102	36.37	73.33	202,659
Tuebingen	GRCz10 (reference)	1,340,447,187	54,191,831	11	36.62	137.39	3,399

Table S7

Genomic scaffolds associated with zebrafish chromosome 8. Scaffolds were identified using BLAST searches for Discovar assemblies from the CG2, CG1, the AB3 individual zebrafish genomes.

Haplotype	GRCz10	CG1	CG2	AB3
mhc2dab, *mhc2daa*	CU694380.6	flattened_line_39082	flattened_line_39346	NA
mhc2dgb, *mhc2dga*	CU929676.6	NA	NA	flattened_line_40646

Table S8

Nomenclature and expression for zebrafish MHC class II genes. Expression data was adapted from 'Sequencing the Zebrafish transcriptome from a range of tissues and developmental stages' (https://www.ncbi.nlm.nih.gov/bioproject/PRJEB1986/). Expression is shown in Reads Per Kilobase of transcript, per Million mapped reads (RPKM). 'Mean RPKM' provides a rough estimate and comparison of expression levels from a female zebrafish head.

Full name	Short name	Historical Name	Chr.	Mean RPKM	Refseq ID	ZFIN ID	Ensembl Gene
mhc2daa	daa	si:busm1-266f07.2	8	148.263	NP_001004521.3	ZDB-GENE-030616-436	ENSDARG00000031745
mhc2dab	dab	si:dkeyp-2h4.1	8	231.094	NP_571551.3	ZDB-GENE-980526-200	ENSDARG00000079105
mhc2dga	dga	si:busm1-194e12.11	8	89.689	NP_001007206.1	ZDB-GENE-030616-505	ENSDARG00000103716
mhc2dgb	dgb	si:busm1-194e12.12	8	86.839	NP_001005943.3	ZDB-GENE-030616-319	ENSDARG00000104635
mhc2dba	dba	si:busm1-241h12.2p	18	1.257	XR_001796469.2	ZDB-GENEP-030616-407	ENSDARG00000093885
mhc2dbb	dbb	si:busm1-241h12.1	18	2.536	NP_001070245.1	ZDB-GENE-010112-2	ENSDARG00000056330
mhc2dca	dca	zmp:0000000884	8	0.841	XP_009302501.2	ZDB-GENE-130530-887	ENSDARG00000114601
mhc2dcb	dcb	si:busm1-228j01.4	8	0.158	NP_001009597.1	ZDB-GENE-030616-394	ENSDARG00000088872

Table S9

Zebrafish and Human MHC genes, and Human NLR genes

Major Histocompatibility Complex (MHC) and NOD-like receptor (NLR) gene lists were compiled from annotated gene lists for each species, and used to identify exon overlap with ZCRs, based on canonical Ensembl gene models. 'Dr' refers to zebrafish (Danio rerio), and 'Hs' refers to human (Homo sapiens). The zebrafish NLR gene set was based on published reference genome annotation efforts (Howe et al. 2016) for genes remaining in Ensembl v85.

Species	Gene set	Ensembl identifier	Gene name
Dr	MHC	ENSDARG00000001470	mhc1zea
Dr	MHC	ENSDARG00000069471	mhc1zca
Dr	MHC	ENSDARG00000036588	mhc1zba
Dr	MHC	ENSDARG00000088022	mhc1zfa
Dr	MHC	ENSDARG00000092162	mhc1zaa
Dr	MHC	ENSDARG00000074765	mhc1zja
Dr	MHC	ENSDARG00000055813	si:dkey-225f5.5
Dr	MHC	ENSDARG00000016056	mhc1laa
Dr	MHC	ENSDARG00000016227	CU571323.1
Dr	MHC	ENSDARG00000092731	mhc1uka
Dr	MHC	ENSDARG00000075963	mhc1uba
Dr	MHC	ENSDARG00000039164	mhc1uma
Dr	MHC	ENSDARG00000059039	mhc1ula
Dr	MHC	ENSDARG00000097275	si:ch211-147g22.4
Dr	MHC	ENSDARG00000051710	CR339041.1
Dr	MHC	ENSDARG00000096830	mhc1lja
Dr	MHC	ENSDARG00000051711	CR339041.2
Dr	MHC	ENSDARG00000051712	mhc1lfa
Dr	MHC	ENSDARG00000023203	mhc1lda
Dr	MHC	ENSDARG00000096977	mhc1lla
Dr	MHC	ENSDARG00000096940	arpc1a
Dr	MHC	ENSDARG00000051713	mhc1lga
Dr	MHC	ENSDARG00000097766	mhc1lia
Dr	MHC	ENSDARG00000104293	mhc1zka
Dr	MHC	ENSDARG00000101337	zgc:103700
Dr	MHC	ENSDARG00000074510	si:busm1-104n07.3
Dr	MHC	ENSDARG00000104635	si:busm1-194e12.12
Dr	MHC	ENSDARG00000076307	si:zfos-2070c2.3
Dr	MHC	ENSDARG00000104730	si:dkey-33b17.3
Dr	MHC	ENSDARG00000101030	si:ch73-158p21.3
Dr	MHC	ENSDARG00000041705	si:busm1-228j01.6

Dr	MHC	ENSDARG00000056330	mhc2dbb
Dr	MHC	ENSDARG00000088872	si:busm1-228j01.4
Dr	MHC	ENSDARG00000104317	si:busm1-160c18.1
Dr	MHC	ENSDARG00000086294	CU914776.1
Dr	MHC	ENSDARG00000031745	si:busm1-266f07.2
Dr	MHC	ENSDARG00000103716	si:busm1-48c11.3
Dr	MHC	ENSDARG00000075932	si:ch73-211l2.3
Dr	MHC	ENSDARG00000074816	si:zfos-367g9.1
Dr	MHC	ENSDARG00000070206	si:zfos-2070c2.1
Dr	MHC	ENSDARG00000101675	zgc:123107
Dr	MHC	ENSDARG00000103702	si:busm1-194e12.8
Hs	MHC	ENSG00000160862	AZGP1
Hs	MHC	ENSG00000166710	B2M
Hs	MHC	ENSG00000158477	CD1A
Hs	MHC	ENSG00000158485	CD1B
Hs	MHC	ENSG00000158481	CD1C
Hs	MHC	ENSG00000158473	CD1D
Hs	MHC	ENSG00000158488	CD1E
Hs	MHC	ENSG00000104870	FCGRT
Hs	MHC	ENSG00000010704	HFE
Hs	MHC	ENSG00000206503	HLA-A
Hs	MHC	ENSG00000234745	HLA-B
Hs	MHC	ENSG00000204525	HLA-C
Hs	MHC	ENSG00000204257	HLA-DMA
Hs	MHC	ENSG00000242574	HLA-DMB
Hs	MHC	ENSG00000204252	HLA-DOA
Hs	MHC	ENSG00000241106	HLA-DOB
Hs	MHC	ENSG00000231389	HLA-DPA1
Hs	MHC	ENSG00000223865	HLA-DPB1
Hs	MHC	ENSG00000196735	HLA-DQA1
Hs	MHC	ENSG00000237541	HLA-DQA2
Hs	MHC	ENSG00000179344	HLA-DQB1
Hs	MHC	ENSG00000232629	HLA-DQB2
Hs	MHC	ENSG00000204287	HLA-DRA
Hs	MHC	ENSG00000196126	HLA-DRB1
Hs	MHC	ENSG00000198502	HLA-DRB5
Hs	MHC	ENSG00000204592	HLA-E
Hs	MHC	ENSG00000204642	HLA-F
Hs	MHC	ENSG00000204632	HLA-G
Hs	MHC	ENSG00000204520	MICA

Hs	MHC	ENSG00000204516	MICB
Hs	MHC	ENSG00000153029	MR1
Hs	MHC	ENSG00000164520	RAET1E
Hs	MHC	ENSG00000203722	RAET1G
Hs	MHC	ENSG00000155918	RAET1L
Hs	MHC	ENSG00000111981	ULBP1
Hs	MHC	ENSG00000131015	ULBP2
Hs	MHC	ENSG00000131019	ULBP3
Hs	NLR	ENSG00000179583	CIITA
Hs	NLR	ENSG00000249437	NAIP
Hs	NLR	ENSG00000167984	NLRC3
Hs	NLR	ENSG00000091106	NLRC4
Hs	NLR	ENSG00000140853	NLRC5
Hs	NLR	ENSG00000091592	NLRP1
Hs	NLR	ENSG00000022556	NLRP2
Hs	NLR	ENSG00000215174	NLRP2B
Hs	NLR	ENSG00000162711	NLRP3
Hs	NLR	ENSG00000277883	NLRP3P1
Hs	NLR	ENSG00000160505	NLRP4
Hs	NLR	ENSG00000171487	NLRP5
Hs	NLR	ENSG00000174885	NLRP6
Hs	NLR	ENSG00000167634	NLRP7
Hs	NLR	ENSG00000179709	NLRP8
Hs	NLR	ENSG00000185792	NLRP9
Hs	NLR	ENSG00000256581	NLRP9P1
Hs	NLR	ENSG00000182261	NLRP10
Hs	NLR	ENSG00000179873	NLRP11
Hs	NLR	ENSG00000142405	NLRP12
Hs	NLR	ENSG00000173572	NLRP13
Hs	NLR	ENSG00000158077	NLRP14
Hs	NLR	ENSG00000160703	NLRX1
Hs	NLR	ENSG00000106100	NOD1
Hs	NLR	ENSG00000167207	NOD2

*Howe K, Schiffer PH, Zielinski J, Wiehe T, Laird GK, Marioni JC, Soylemez O, Kondrashov F, Leptin M. 2016. Structure and evolutionary history of a large family of NLR proteins in the zebrafish. *Open Biol* **6**: 160009.

Table S10: Summary of ZCR regions and number of genes found overlapping with ZCRs for the zebrafish and human genomes.

Sample	Total Regions	Median Size (bp)	Max Size (bp)	Total length (GB)	Genome (%)	Genes with ≥1 exon in ZCR
CHM	236	3325	36450	1.08	0.04	15
CEU	163	3050	11200	0.57	0.02	12
YRI	79	2800	9150	0.26	0.01	8
CG2	3784	2850	20900	13.35	1	632
CG1	6214	2850	49200	24.5	1.83	1081
AB3	3219	2900	49950	13.38	1	538
